# Beyond the blood–brain barrier: unraveling T cell subsets in CNS immunity and disease

**DOI:** 10.1007/s10787-025-01955-z

**Published:** 2025-09-24

**Authors:** Mustafa M. Shokr

**Affiliations:** Department of Pharmacology and Toxicology, Faculty of Pharmacy, Sinai University – Arish Branch, Arish, 45511 Egypt

**Keywords:** T-cell subsets, Neuroinflammation, Neurodegeneration, Immunomodulation

## Abstract

The complicated interplay between the central nervous system (CNS) and immune system is critical for both neuroprotection and neurodegeneration. T lymphocytes are key players in CNS immunity, with distinct subgroups that work in a highly dynamic and, sometimes, antagonistic manner depending on the disorder. This review covers T-cell subgroups with a focus on pathogenic Th1 and Th17 cells and their role in mediating neuronal death and demyelination in multiple sclerosis (MS) and stroke, as well as the neurotoxic nature of CD8+ cytotoxic T lymphocytes and the neuroprotective and immunosuppressive roles of regulatory T cells (Tregs) that promote CNS homeostasis. In answer to the overwhelming need for effective pharmacotherapy, we overview of available pharmacotherapies that have the potential to target the aforementioned T-cell subsets. Treatments ultimately include broad-spectrum immunosuppressants, highly specific monoclonal antibodies, and new small-molecule inhibitors. Clinical data are added whenever possible to assess how these therapies interact with T-cell activity to restore immune balance in the CNS. This review highlights the evolution of treatment progress and the growing paradigm shift towards precision medicine through the targeting of T-cell subsets in neuroinflammatory diseases.

## Introduction

### Neuroinflammation: a double-edged sword in CNS disorders

The complex immune response of the central nervous system (CNS) to many stimuli, such as infection, trauma, and the accumulation of abnormal proteins that signify neurodegenerative diseases (NDDs), is known as neuroinflammation (Xu et al. 2025). This intricate process is most frequently described as a “double-edged sword” because of its inherent duality: it is most dangerous when dysregulated or chronic, yet it may be a vital defense mechanism when it is protective and acute (Adamu et al. 2024; Xu et al. 2025). In its protective, acute phase, neuroinflammation guides the recruitment of immune cells, specifically resident microglia and astrocytes, to eliminate pathogens, eliminate cellular waste, and initiate repair processes. For instance, activated microglia take up misfolded proteins such as α-synuclein in Parkinson’s disease (PD) or amyloid-beta in Alzheimer’s disease (AD) (Adamu et al. 2024).

This defense mechanism typically turns into a persistent, unresolved “state of affairs” of low-level, but extremely damaging, inflammation in NDDs. This unresolved neuroinflammation is caused by the continuously activated glial cells, which emit reactive oxygen and nitrogen species as well as pro-inflammatory cytokines, tumor necrosis factor alpha (TNF-α), interleukin-1 beta (IL-1β), and IL-6 over a prolonged period (Dash et al. 2025). Instead of promoting repair, this chronic inflammatory milieu exacerbates protein aggregation, neuronal malfunction, and death, and the progressive nature of NDDs such as AD, PD, and multiple sclerosis (MS) (Dash et al. 2025). The continuous production of inflammatory mediators and glial phenotypes (such as M1-like microglia and A1-like astrocytes) that inhibit neurogenesis, disrupt synaptic organization, and compromise the integrity of the blood–brain barrier (BBB) also contributes to a cycle of neurodegeneration (Deng et al. 2024; Badawi et al. 2024). Therefore, a clear knowledge of the delicate balance and mechanisms underlying the transition from protective to destructive neuroinflammation is necessary for the development of effective therapeutic approaches for such severe illnesses (Deng et al. 2024).

### Overview of T lymphocytes and subsets

T cells, also known as T lymphocytes, are essential components of the adaptive immune system that coordinate a highly specialized and varied array of immunological responses that are critical for the host's defense against malignancies and infections (Wang et al. 2024). Hematopoietic stem cells produced from bone marrow undergo a complex maturation process involving both positive and negative selection, resulting in T cell precursors that move to the thymus. The procedure guarantees that a variety of T cell receptors (TCRs) are developed to identify specific antigens that are presented by major histocompatibility complex (MHC) molecules (Wang et al. 2024). This antigen-specific recognition, which allows T cells to provide targeted responses against a variety of threats without losing tolerance to self-antigens, is what defines adaptive immunity. When activated by an antigen-presenting cell (APC) exhibiting a relevant peptide-MHC complex, naive T lymphocytes proliferate and differentiate into discrete effector and memory subsets. Specific surface markers, cytokine profiles, and functional roles set these subsets apart (Chi et al. 2024).

Because it enables a tailored immune response to infections or disease states, T cell functional heterogeneity is crucial. One main lineage, CD4+ T helper (Th) cells, is essential for coordinating immune responses because they release a variety of cytokines that impact other immune cells (Raphael et al. 2014). Th1 cells produce interferon-gamma (IFN-γ) and TNF-α, which are hallmarks of cell-mediated immunity to intracellular infections. They also play a role in delayed-type hypersensitivity reactions and inflammation (Raphael et al. 2014).

By secreting the interleukins IL-4, IL-5, and IL-13, Th2 cells, on the other hand, play a significant role in humoral immunity, organizing defenses against extrinsic parasites, and significantly influencing allergic reactions (Kokubo et al. 2022). Although Th17 cells, which have been more recently identified and express IL-17 and IL-22, are also necessary for the host's defense against external bacteria and fungi, they are also strong inducers of inflammatory and autoimmune disorders due to their strong pro-inflammatory cytokine profile (Zambrano-Zaragoza et al. 2014).

One important CD4+ fraction known as regulatory T cells (Tregs) is distinguished by the expression of the transcription factor FoxP3. Tregs contribute to immunological homeostasis, anti-autoimmunity, and the suppression of inflammatory responses by firmly suppressing effector T cell activation and proliferation, as well as other immune cells (Kitagawa et al. 2013). Apart from CD4+ T cells, CD8+ cytotoxic T lymphocytes (CTLs) are the second significant lineage. Granzymes and perforin, which are secreted by activated CTLs, induce apoptosis and destroy target cells, including cancerous and virus-infected cells, directly (Kitagawa et al. 2013). These multifunctional T cell subsets, with their specialized function and intricate interactions, form the basis of adaptive immunity, which enables the immune system to provide efficient and controlled responses to a wide range of threats, even though dysregulation in their balance or function has the potential to cause immunodeficiency, autoimmune disease, or inflammatory disease (Kitagawa et al. 2013).

The expanding understanding of the roles of specific T cell subsets in neurodegenerative and neuroinflammatory diseases will be discussed in this review. We will present the pathogenic functions of pro-inflammatory T cells and contrast them with the regulatory subsets’ immunomodulatory and neuroprotective functions. Finally, we will discuss new treatment strategies that use T-cell regulation to treat disorders of the central nervous system. In Table 1, the various T-cell subsets, their characteristics, and their roles in CNS immunity and disease are presented.

**Table 1:** Summary of the various T-cell subsets, their characteristics, and their roles in CNS immunity and disease

T-cell subset	Key characteristics/markers	Pathogenic role in CNS	Associated CNS diseases	Refs.
Th1	IFN-γ	Promotes inflammation, activates microglia, BBB disruption	Multiple sclerosis (MS), stroke, AD, PD	Li et al. (2015), Qin et al. (2022)
Th17	IL-17	Induces inflammation, tissue damage, and BBB disruption	MS, stroke, AD, PD	Qiu et al. (2021), Shi et al. (2022)
CD8+ cytotoxic T lymphocytes	CD8, granzymes, perforin	Direct neuronal cytotoxicity, demyelination	MS, viral encephalitis	Atkinson et al. (1998)
Regulatory T cells (Tregs)	FoxP3, CD25	–	MS, stroke, AD, PD, amyotrophic lateral sclerosis	Terrabuio et al. (2023)
Th9	IL-9	Contributes to neuroinflammation	MS	Elyaman and Khoury (2017)
Th22	IL-22	Promotes inflammation, tissue damage	MS	Goverman (2009)
Tissue-resident memory T cells	CD69, CD103	Can contribute to chronic inflammation in the CNS	MS, AD, encephalitis	Bettini and Vignali (2010)

## T-cell entry and traffic into the CNS

### The BBB and blood-cerebrospinal fluid barrier

Intricate interfaces known as the BBB and the blood-cerebrospinal fluid (CSF) barrier (BCSFB) enable the CNS to preserve a tightly regulated internal environment, which is essential for its sensitive neuronal function (Engelhardt and Sorokin 2009).

The barriers act as strong physical and metabolic gatekeepers, tightly controlling the movement of molecules, ions, and cells from systemic circulation to the neural parenchyma or CSF. This protects the brain from harmful substances, immune cell penetration, and changing peripheral conditions (Engelhardt and Sorokin 2009; Abulaban et al. 2025). The cerebral capillaries are the primary location of the BBB, a complex neurovascular unit composed of several cooperating cellular components. The highly specialized brain microvascular endothelial cell serves as its anatomical basis (Hawkins and Davis 2005). One of the distinctive features that sets brain microvascular endothelial cells (BMECs) apart from peripheral endothelial cells is the presence of massive tight junctions (TJs), which significantly limit paracellular diffusion (Cui et al. 2013). These TJs, which are made up of transmembrane proteins like as occludin, claudins (particularly claudin-5), and junctional adhesion molecules, create a continuous seal between neighboring endothelial cells. The zona occludin (ZO) proteins (ZO-1, ZO-2, and ZO-3) bind these TJs to the actin cytoskeleton (Cui et al. 2013). Adheren junctions (AJs), mostly composed of vascular endothelial (VE)-cadherin, are less restrictive than TJs but also improve barrier integrity and endothelial cell adhesion (Wallez and Huber 2008). The BBB is induced and maintained by pericytes, which are encased in the basement membrane and surround the BMECs. They regulate capillary blood flow, immune cell entrance, and the growth, development, and survival of endothelial cells (Winkler et al. 2021). Since astrocyte end-feet are extensions of astrocytes, they occupy approximately 99% of the capillary surface that is devoid of pericytes. By regulating food transit, buffering ion levels, and secreting substances that support TJ formation and function, they preserve the integrity of the BBB (Abulaban et al. 2025).

Last but not least, neurons, microglia, and the extracellular matrix support the neurovascular unit, collaborating to create the unique environment needed for the best possible neuronal function (Abulaban et al. 2025). In addition to this physical barrier, the BBB achieves this by controlling molecule motion through an intricate network of transport mechanisms and enzymatic activities (Aborode et al. 2025).

Certain transporters, such as glucose transporter 1, allow for the controlled uptake of vital nutrients like glucose, while efflux pumps, especially the ATP-binding cassette transporters like P-glycoprotein, breast cancer resistance protein, and multidrug resistance-associated protein families, actively pump a variety of xenobiotics, medications, and waste products back into the bloodstream, barring their accumulation in the brain (Löscher and Potschka 2005). Furthermore, before potentially harmful compounds enter the brain, they can be broken down and removed by certain metabolic enzymes found in BMECs (Löscher and Potschka 2005). Under normal circumstances, this multi-layered protective mechanism efficiently blocks the entry of circulating pathogenic pathogens, toxic immune cells, and other plasma elements, maintaining the CNS’s immuno-privileged status (Wu et al. 2023). The BCSFB, which is found at the choroid plexuses inside the brain's ventricles, performs the same gatekeeping between the blood and the CSF (Ghersi-Egea et al. 2018).

Specialized choroid plexus epithelial cells, connected by TJs, secrete the majority of the CSF and filter chemicals from the blood, controlling the content and flow of CSF (Ghersi-Egea et al. 2018). The BCSFB, an active immunological barrier that controls the entry of soluble material and immune cells into the CSF, provides an additional line of defense for the CNS, although it is physically different from the BBB (Engelhardt and Sorokin 2009). The maintenance of CNS homeostasis, peripheral immune cell exclusion, and neuronal function all depend on the BBB and BCSFB working together (Engelhardt and Sorokin 2009).

### T-cell transmigration mechanisms in disease

Under normal conditions, the BCSFB and BBB's impermeable barrier properties prevent T cells and other peripheral immune cells from entering the body (Marchetti and Engelhardt 2020). But in the setting of CNS inflammation, injury, or illness, these barriers are frequently broken, allowing T cell transmigration to occur, which can have a significant impact on the course of the illness (Marchetti and Engelhardt 2020). This complex, multi-step process of T-cell penetration involves dynamic interchange between activated T cells, activated VE, and the CNS (Alcaide 2022). Whether the first insult to the CNS is an ischemic injury (stroke), MS, or AD, it typically sets off a pro-inflammatory response (Alcaide 2022). The majority of inflammatory mediators are produced by activated resident glial cells, such as microglia and astrocytes, and include the cytokines TNF-α, IL-1β, and IL-6 as well as the chemokines CCL2 (MCP-1), CXCL10 (IP-10), and CCL5 (Ramesh et al. 2013). When these inflammatory stimuli come into direct contact with brain endothelial cells, they experience a significant phenotypic change (Ramesh et al. 2013).

On the endothelial cells’ luminal surface, the activation increases the expression of adhesion molecules. Important adhesion molecules called selectins, like P-selectin and E-selectin, attach to carbohydrate T-cell ligands and aid in T-cell rolling and capture (Reglero-Real et al. 2016). The first low-affinity link slows the T cells down as they move through the capillaries, making space for subsequent, higher-affinity contacts (Reglero-Real et al. 2016). The next crucial step is T-cell adhesion to the activated endothelium, which can only be achieved by T-cell surface integrins attaching to immunoglobulin superfamily adhesion molecules on the endothelium (Sökeland and Schumacher 2019). The most potent of these are lymphocyte function-associated antigen-1 on T cells, which interacts with intercellular adhesion molecule-1 on endothelial cells, and Very Late Antigen-4 (VLA-4) on T cells, which interacts with vascular cell adhesion molecule-1 (VCAM-1) on endothelial cells (Van Kooyk et al. 1993).

Prolonged exposure to pro-inflammatory cytokines and chemokines also has a direct impact on the close BMEC connections (Yang et al. 2022). Inflammatory signals that induce TJ proteins (including occludin, claudin-5, and ZO-1) to become phosphorylated, ubiquitinated, and degraded also promote disruption of the paracellular barrier (Cai et al. 2017). Furthermore, the barrier is destabilized and T-cell extravasation is predicted when the matrix metalloproteinases MMP-2 and MMP-9, which are released by activated glia, invasive immune cells, or even endothelial cells themselves, cleave basement membrane and extracellular matrix molecules (such as collagen and laminin) (Lakhan et al. 2013). After adhering to the artery wall, T lymphocytes go through diapedesis and move through the endothelium layer either paracellularly, which is between cells, or transcellularly, which is perpendicular to the cell body (Lakhan et al. 2013). This transmigration is typically guided by chemokine gradients that are released by activated glia in the CNS parenchyma or displayed on the luminal surface of endothelial cells. In MS, for example, certain chemokine axes draw Th1 and Th17 cells into the CNS, resulting in demyelination and neurodegeneration (Ashhurst et al. 2014). The degree and rate of T-cell infiltration are significantly influenced by the type and severity of the inflammatory stimuli, the BBB/BCSFB functional state, and the disease environment. The BBB/BCSFB is a therapeutic target candidate and at the forefront of neuroinflammation control due to this complex series of cellular and molecular events (Ashhurst et al. 2014).

### T-cell surveillance within the healthy CNS

Since the CNS is mostly impermeable to peripheral immune cells and does not rely on systemic immunological pathways, it was formerly thought to be an “immune-privileged” organ due to the strong barrier activities of the BBB and BCSFB (Louveau et al. 2015). This was justified by the finding that allografts remain in the brain for a considerable amount of time (Louveau et al. 2015). However, growing evidence that peripheral T cells are continuously monitoring the intact CNS in a regulated manner has changed this historic perspective slightly (Zang et al. 2022). This surveillance is essential for the immediate detection of infection or cellular abnormalities, enabling a suitable and localized immune response when required, without endangering the delicate brain architecture (Guerrero and Sicotte 2020). We already know that T cells and other leukocytes, including monocytes and dendritic cells, tend to traverse the boundaries of the CNS and CSF compartments when there is no obvious inflammation (Guerrero and Sicotte 2020).

**Fig. 1:**
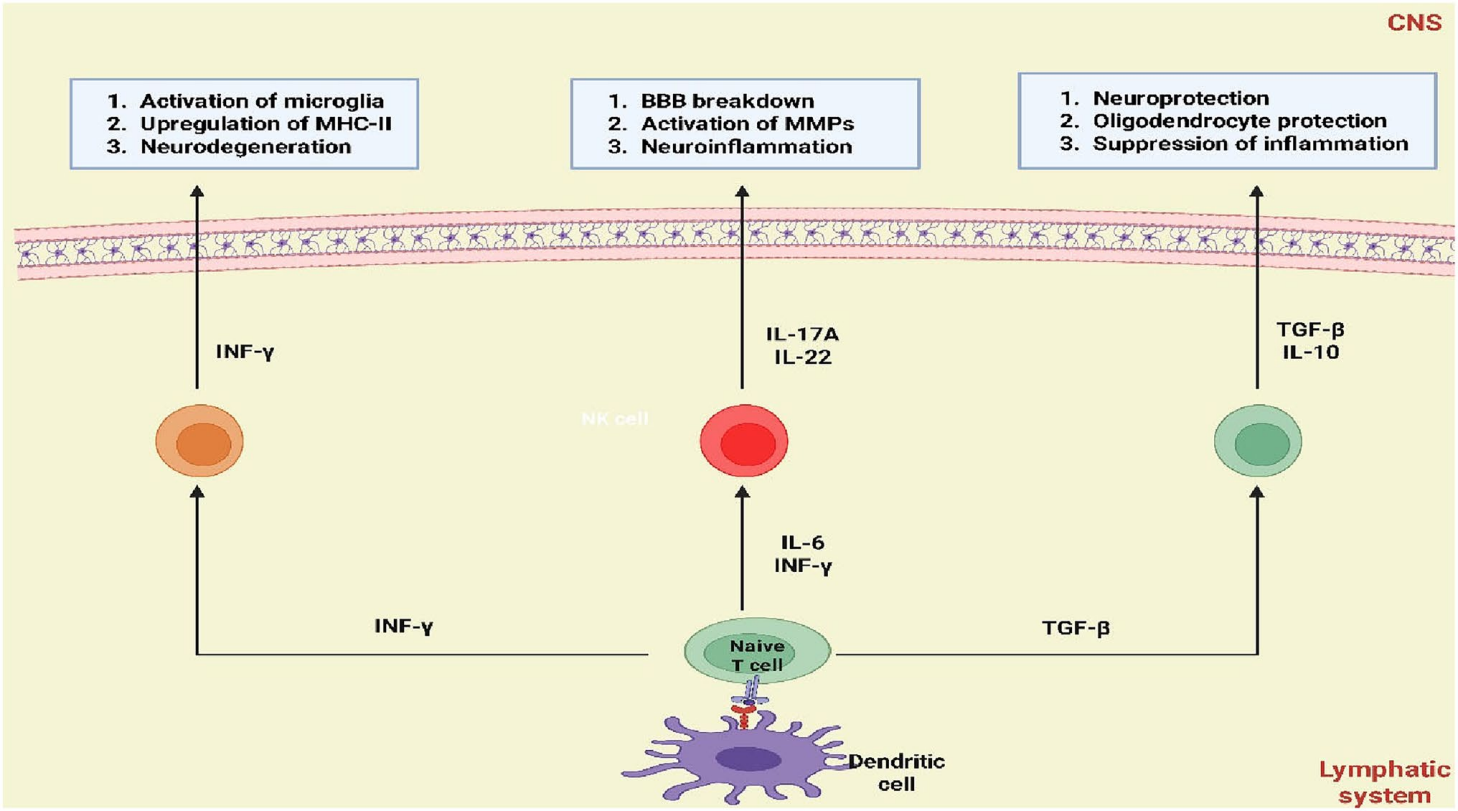
Differential impact of T-cell subsets on central nervous system (CNS) homeostasis. This diagram illustrates the distinct roles of various T-cell subsets in the CNS, mediated by their characteristic cytokine profiles. Interferon-gamma (INF-γ) produced by activated T cells contributes to the activation of microglia, upregulation of major histocompatibility complex-II (MHC-II), and neurodegeneration. Interleukin-17A (IL-17A) and IL-22 lead to blood–brain barrier (BBB) breakdown, activation of Matrix Metalloproteinases (MMPs), and neuroinflammation. Conversely, transforming growth factor-beta (TGF-β) and Interleukin-10 (IL-10) released by regulatory T cells promote neuroprotection, oligodendrocyte protection, and suppression of inflammation, maintaining CNS integrity. The interplay between T cells, dendritic cells, and the lymphatic system influences these outcomes

Choroid plexus, leptomeninges, and even specific perivascular areas next to CNS blood arteries are some of the CNS-draining sites where such physiological trafficking usually occurs (Vara-Pérez and Movahedi 2025). The nature of T-cells in the normal CNS must be distinguished (Vara-Pérez and Movahedi 2025). Although intermittent transient entry and exit suggest a patrolling or surveillance function, the idea that the CNS parenchyma contains a completely "resident" cell population of T-cells in the normal, undisturbed brain is controversial (Smorodchenko et al. 2007). It has been discovered that steady-state populations of memory T cells, particularly those with specific chemokine receptors, are present in the choroid plexus and meninges, which particular subsets of T cells preferentially home to in healthy individuals. This suggests a sentry function prepared to react swiftly to CNS injury (Gerganova et al. 2022) (Fig. 1).

Furthermore, the identification of a functional meningeal lymphatic system that is directly related to cervical lymph nodes has totally changed our knowledge of how immune cells and antigens are transported from the central nervous system and delivered to the peripheral immune system. Rather than complete immunological isolation, this observation is consistent with continuous immune communication (Li et al. 2022). By providing a direct conduit for antigen presentation to T cells in the draining lymph nodes, this lymphatic system facilitates the production of adaptive immune responses to CNS-derived antigens that can re-enter the CNS upon inflammation (Li et al. 2022). Although the brain is well protected, T-cells’ continuous low-level surveillance means that it is not entirely isolated from the systemic immune system (Galea 2021). Under normal physiological conditions, this homeostatically limited immune presence guarantees the quick but regulated activation of an immune response in the event of a real threat without resulting in diffuse inflammation (Galea 2021). Understanding these homeostatic processes is essential for distinguishing between pathological neuroinflammation and physiological immune participation, as well as for developing therapies that can restore rather than eliminate typical immune interactions with the CNS.

## Pathogenic T-cell subsets in neuroinflammatory and neurodegenerative diseases

### Th1 cells: IFN-γ-mediated inflammation

As the primary cells in charge of coordinating cell-mediated immunity, Th1 cells are a vital fraction of CD4+ T lymphocytes and are especially effective against tumor cells and intracellular infections (Ahmed et al. 2022). Both acute and chronic CNS neuroinflammatory illnesses are significantly influenced by their prototypical cytokine, IFN-γ, a strong pro-inflammatory cytokine with several functions in the CNS (Ahmed et al. 2022). Th1 cells and released IFN-γ are essential for activating resident immune cells in the CNS, including astrocytes and microglia. By directly targeting microglia, IFN-γ causes them to adopt a classical (M1-like) pro-inflammatory phenotype, which is characterized by elevated MHC-II molecule and the release of a series of inflammatory mediators, including reactive oxygen species, nitric oxide, TNF-α, and IL-1β (Valiukas et al. 2025). A cytotoxic microenvironment is also produced by the persistent microglia activation, which promptly results in neuronal damage and death (Valiukas et al. 2025). In addition, IFN-γ directly impairs neuronal function, causes neuronal death, and damages oligodendrocytes, which results in demyelination (Valiukas et al. 2025). IFN-γ can destabilize endothelial cell tight junctions, enhance barrier permeability, and further encourage peripheral immune cell invasion into the CNS parenchyma and activation of the inflammatory cascade, all of which have a profound impact on the permeability of the BBB (Angelini et al. 2023).

In a number of neuroinflammatory and neurodegenerative disorders, pathogenic Th1 cells and IFN-γ are implicated. Th1 cells have historically been thought to be the first to cause autoimmune demyelination and axonal damage in MS (Angelini et al. 2023). Th1 cells continue to play a significant role in MS pathogenesis, aiding in the development of lesions and the advancement of the illness, even though their function has been expanded to include Th17 cells (Angelini et al. 2023). Th1 cells cause subsequent brain injury in ischemic stroke by penetrating the ischemic core and penumbra. These cells release IFN-γ, which may promote inflammation, BBB permeability, and neuronal death, resulting in a bigger infarct and poorer functional recovery (Qin et al. 2022). According to recent data, neurological illnesses, including PD and AD, are also influenced by Th1 responses. The peripheral blood and autopsy brains of AD patients have been found to contain larger percentages of Th1 cells and elevated amounts of IFN-γ (Qin et al. 2022).

Additionally, it has been proposed that IFN-γ has a role in the development of amyloid-beta (Aβ) plaque and tau hyperphosphorylation (Li et al. 2015). This could be through mechanisms that directly impact neuronal pathology or by causing persistent microglial activation that impedes Aβ clearance (Li et al. 2015). Similarly, in PD, the neuroinflammatory processes that lead to the death of dopaminergic neurons have been connected to an increase in T cells that produce IFN-γ (Xu et al. 2023). Evidence suggests that uninhibited or chronic Th1 responses contribute significantly to the pathological features of neuroinflammation, amplifying neuronal injury and promoting disease pathology in a variety of CNS disorders, even though the exact interaction is complicated and possibly context-dependent (Xu et al. 2023).

### Th17 cells: IL-17 and autoimmunity

Th17 cells are a powerful pro-inflammatory subset of CD4+ T cells that are distinguished by the secretion of hallmark cytokines, namely interleukin-17A (IL-17A), IL-17F, IL-21, and IL-22. TGF-β, IL-6, and IL-21 are crucial for Th17 cell development, which is sustained by IL-23 (Brockmann et al. 2017). Since their discovery, Th17 cells have become important mediators of autoimmune and inflammatory diseases, especially at mucosal interfaces and in chronic inflammation (Brockmann et al. 2017). They have strong pro-inflammatory properties because they can cause various cell types, such as fibroblasts, epithelial cells, endothelial cells, and glial cells, to produce cytokines (like IL-6 and TNF-α), chemokines (like CCL2, CXCL1, and CXCL8), and matrix metalloproteinases (MMPs) (Wang et al. 2023). This leads to neutrophils and macrophages actively invading the tissue (Shi et al. 2022; Wang et al. 2023). Th17 cells cause significant tissue damage in the central nervous system and play a key role in the processes of neuroinflammation and neurodegeneration (Shi et al. 2022). Their only significant mechanism is their direct effect on the integrity of the BBB; IL-17 directly destroys brain endothelial cell TJs, increasing BBB permeability. Local inflammation is exacerbated and contributed to by this breach, which makes it easier for peripheral immune cells, including other pathogenic T cells, to migrate into the CNS parenchyma (Shi et al. 2022). Additionally, Th17-derived cytokines can trigger the synthesis of MMP by endothelial cells and astrocytes, which further breaks down the extracellular matrix and basement membrane components of the neurovascular unit, disrupting the BBB and making neurons more vulnerable (Qiu et al. 2021).

Th17 cells are thought to have a major role in the pathophysiology of autoimmune demyelination and neurodegeneration, which is arguably best demonstrated in autoimmune CNS diseases such as MS (Zheng and Luo 2025). Th17 cells are becoming more and more linked to the etiology of neuroinflammatory diseases outside of MS. Th17 cells quickly proliferate in the brain after an ischemic stroke, and because they may attract neutrophils and secrete pro-inflammatory cytokines, they are linked to acute inflammation and worsening of neuronal injury (Wang et al. 2023). Increased Th17 cell and IL-17 frequencies and levels have been observed in the brains and periphery of AD patients, which may be linked to astrocyte reactivity (e.g., causing the neurotoxic A1-like phenotype), microglial activation, and the exacerbation of amyloid pathology (Fu et al. 2022). Similar to this, PD has been linked to a changed Th17 cell to Treg ratio, and elevated Th17 cells may be a factor in dopaminergic neuron degeneration (Fu et al. 2022).

### CD8 + cytotoxic T lymphocytes: direct neurotoxicity

Cytotoxic T lymphocytes (CTLs), also known as CD8+  T lymphocytes, are a unique and extremely specialized subset of T cells that all have the same main objective of identifying and eliminating target cells (Sykulev 2023). CTLs can kill cells by causing apoptosis in cells that express foreign or abnormal antigens on major MHC-I molecules, in contrast to CD4+ T helper cells, which mainly release cytokines to coordinate immune responses (Sykulev 2023). Two important pathways are the main ways that CTLs cause their cytolytic. The first and best-known of these is the perforin/granzyme pathway, in which CTLs release pre-formed granules containing perforin and other granzymes when they encounter a target cell (Atkinson et al. 1998). Granzymes (such as Granzyme A and Granzyme B) enter the cytoplasm through the holes that perforin creates in the target cell membrane. Granzymes in the cytoplasm set off subsequent apoptotic cascades that result in the targeted cell’s deliberate demise (Goping et al. 2003). The second main mechanism is the Fas/Fas ligand (FasL) route, in which target cells' Fas receptor recognizes the surface expression of FasL by activated CTLs. The extrinsic apoptotic cascade, which involves caspases and results in programmed cell death, is started by this contact (Volpe et al. 2016).

CD8+ CTLs have a variety of uses and occasionally conflicting roles in the CNS. It is undeniable that they protect against CNS infections, especially viral encephalitis and meningitis (Mockus et al. 2019). For example, CTLs play a crucial role in eliminating infected neurons in HSV encephalitis; nevertheless, their strong cytotoxicity can also result in significant immunopathology and harm to bystander neurons. It is becoming increasingly acknowledged that CD8+ T cells are linked to chronic neuroinflammatory and neurodegenerative disorders in addition to direct infection (Mockus et al. 2019). The most prevalent population in active demyelinating lesions in MS is CD8+ T lymphocytes, which preferentially remain near injured oligodendrocytes and axons (Wootla et al. 2012). Additionally, it has been demonstrated that CTLs can identify oligodendroglial or neuronal antigens displayed on MHC-I and directly cause axonal transection and demyelination. In fact, in certain MS models, CD8+ T cells enter the CNS before CD4+ T cells, suggesting an early and direct pathogenic impact (Wootla et al. 2012). CD8+ T lymphocytes have been implicated in neurodegenerative illnesses such as PD and AD, according to recent findings. There have been reports of higher numbers of clonally amplified CD8+ T lymphocytes in AD brains, especially those with chronic activation or exhaustion characteristics, which challenge their target antigens and contribute to neuronal death (Terrabuio et al. 2023). Likewise, in PD, α-synuclein peptides presented by MHC-I can be recognized by CD8+ T lymphocytes in the substantia nigra, indicating direct cytotoxicity to dopaminergic neurons in people with particular HLA haplotypes (Terrabuio et al. 2023).

Invading CD8+ T lymphocytes generate secondary harm in stroke and traumatic brain injury (TBI), acute CNS trauma by destroying injured cells while simultaneously increasing bystander injury and causing inflammation (Daglas et al. 2019). Research is still being done on the precise triggers for CD8+ T cell activation, their precise targets inside the CNS in such non-infectious situations, and how their pathogenic function of increasing neurotoxicity balances with their protective function of clearing debris.

### Other context-dependent or pathogenic T-cell subsets

The landscape of T-cell immunology is far more diverse outside of the conventionally recognized Th1, Th17, and CD8+ CTLs, and includes a number of other subsets that can act in the CNS in a pathogenic or context-dependent manner (Montauti et al. 2024). The roles of these non-canonical T-cell subsets in neuroinflammation and neurodegeneration are becoming more and more clear, exposing a complicated interaction that can have a big impact on how a disease turns out. They include Th9 cells, which are distinguished by their IL-9 production (Deng et al. 2017; Montauti et al. 2024). Th9 cells were first linked to allergy reactions and helminth host immunity, but they are now also linked to autoimmune disorders and can maintain inflammation (Deng et al. 2017). Th9 cells have been linked to CNS autoimmunity by causing neuroinflammation and demyelination in EAE, the animal model of MS (Elyaman and Khoury 2017). Although their exact roles in human neurodegenerative illnesses are yet unknown, their ability to activate mast cells and attract other inflammatory cells points to a possible pro-inflammatory effect (Elyaman and Khoury 2017).

Similar to this, Th22 cells are traditionally involved in tissue healing and host defense at barrier surfaces. Their main characteristic is the release of IL-22 in the absence of other Th1, Th2, or Th17 signature cytokines (Fujita 2013). Though they have context-dependent neuroprotective benefits, Th22 cells and IL-22 have been found in MS lesions and the CSF fluid of MS patients. They are thought to be involved in the maintenance of inflammation and tissue damage in the CNS (Rolla et al. 2014). Although the exact mechanisms of Th22 cell action in neurodegeneration are still being studied, glial activation or breakdown of the BBB may result from altering the function of epithelial cells and inflammatory processes (Rolla et al. 2014).

Paradoxically, impaired T cells may prevent unchecked autoimmune tissue damage by limiting excessive, chronic immunopathology in the CNS. It may, however, also interfere with the therapeutic removal of harmful protein aggregates or damaged cells in neurodegeneration (Goverman 2009). However, tissue-resident memory T cells, or Trm cells, are becoming more and more common in the CNS (Ayasoufi et al. 2023). These memory cells that are not in circulation are always present in peripheral tissues, possibly even the CNS periphery (choroid plexus, meninges). Trm cells contribute to quick local protective immunity against re-infection, but as sentinels that can quickly turn pathogenic, their permanent establishment and ability to quickly reactivate upon local inflammation may cause chronic neuroinflammation or autoimmune flare-ups in the CNS (Ayasoufi et al. 2023).

## Regulatory T-cell subsets: counteracting neuroinflammation and promoting repair

### Development and definition of Tregs

The major functions of Tregs, a rare but essential subset of CD4+ T lymphocytes, are to preserve immunological homeostasis, inhibit autoimmunity, and reduce excessive inflammatory reactions (Goswami et al. 2022). Constitutive expression of the intracellular transcription factor Forkhead box P3 (FoxP3), a master gene for their development, survival, and suppressive function, is what distinguishes them. The discovery that human mutations in the FOXP3 gene cause IPEX syndrome (immune dysregulation, polyendocrinopathy, enteropathy, and X-linked syndrome), a severe autoimmune disease marked by widespread systemic autoimmunity due to the loss or impairment of Tregs, further supports the critical role of FoxP3 (Sato 2025). According to their origin and line of differentiation, Tregs are often divided into two groups: peripherally induced Tregs (iTregs) and naturally occurring or thymic-derived Tregs (tTregs, also known as nTregs) (Pellerin et al. 2014). Conventional T cells that have a high affinity for self-antigens during T-cell maturation give rise to tTregs in the thymus. In addition to preserving central tolerance and serving as essential sentinels of self-reactivity, this thymic selection mechanism is responsible for the development of a repertoire of Treg cells that are specially programmed to recognize self-peptides (Bettini and Vignali 2010). With a highly activated or “memory-like” phenotype, constitutive expression of CTLA-4, and high expression of the IL-2 receptor α-chain (CD25), these tTregs are frequently exported from the thymus as mature, suppressive cells that have already established themselves in their regulatory phenotype (Povoleri et al. 2013).

As an alternative, iTregs arise from peripheral naive CD4+ FoxP3-T cells, usually following antigenic challenge in the lymphoid organs or inflammatory tissues under certain tolerogenic circumstances (Povoleri et al. 2013). Transforming growth factor-beta (TGF-β), other cytokines or pathways, or the lack of potent pro-inflammatory stimuli are the primary environmental cues for iTregs differentiation (Li and Flavell 2008). Even though both iTregs and tTregs express FoxP3 and have suppressive properties, they may have different TCR repertoires, migratory patterns, and epigenetic markers that allow them to perform context-specific regulatory tasks (Wing et al. 2019). Apart from FoxP3, Tregs are distinguished by the presence of several other intracellular and surface molecules that contribute to their identification and activity. These include elevated levels of TNFR2, CD25, as well as a predominantly anergic state to intense T-cell receptor stimulation unless there is significant co-stimulation (Chen et al. 2010).

### Mechanisms of Treg-mediated immunosuppression in the CNS

To carry out their strong immunosuppressive duties and reduce unwarranted inflammation and preserve immunological homeostasis in a variety of organs, including the immunologically privileged CNS, Tregs use a broad range of mechanisms (Zhang et al. 2024). Their distinct capacity to control neuroinflammation can be explained by their broad classification under the following headings: contact-dependent suppression, cytokine-mediated suppression, metabolic disruption, and modification of APC activity (Zhang et al. 2024).

#### Mechanisms relying on direct contact

Tregs use a variety of inhibitory receptors on their surface to interact with ligands on effector T cells or APCs (Wardell et al. 2021). For example, Tregs constitutively have high levels of lymphocyte Antigen 4 (CTLA-4). Compared to CD28, the effector T-cell co-stimulatory molecule, CTLA-4, binds to CD80 and CD86 (B7 molecules) on APCs with a higher affinity. This competition for CD28 binding causes CD80/CD86 to be downregulated on APCs (Wardell et al. 2021). This causes anergy or death in the cells by blocking co-stimulatory signals that are essential for the best activation and growth of traditional effector T cells (Wardell et al. 2021). Additionally, the activation of CTLA-4 triggers the production of immunomodulatory kynurenine metabolites and the stimulation of indoleamine 2,3-dioxygenase by APCs, an enzyme that catabolizes tryptophan and is required for T-cell proliferation (Massalska et al. 2022). Although it is not a Treg-specific mechanism, Tregs also use the programmed death-1 (PD-1) pathway, in which they generate PD-1 ligands (PD-L1/PD-L2) that interact with PD-1 on effector T cells to inhibit them. As a context-dependent suppressive mechanism, Tregs can also directly cause effector T cells or APCs to undergo apoptosis by secreting granzyme and perforin, a function that is typically given to CD8+ CTLs (Tay et al. 2023).

#### Suppression mediated by cytokines

Tregs are known to produce very immunosuppressive cytokines (Wan 2010). One of the most significant anti-inflammatory cytokines released by Tregs is IL-10, which directly prevents many immune cells, including T cells, macrophages, and microglia, from producing pro-inflammatory cytokines (such as TNF-α, IL-1β, and IFN-γ) (Wan 2010). By suppressing the production of co-stimulatory molecules and MHC class II, IL-10 also reduces APC function (Wan 2010). Tregs also release the pleiotropic cytokine TGF-β, which has a variety of immunosuppressive effects. TGF-β can directly suppress T cell proliferation and effector function, induce FoxP3 expression in iTregs under specific circumstances, and encourage apoptosis in effector T cells (Moreau et al. 2022).

#### Metabolic disruption

**Fig. 2:**
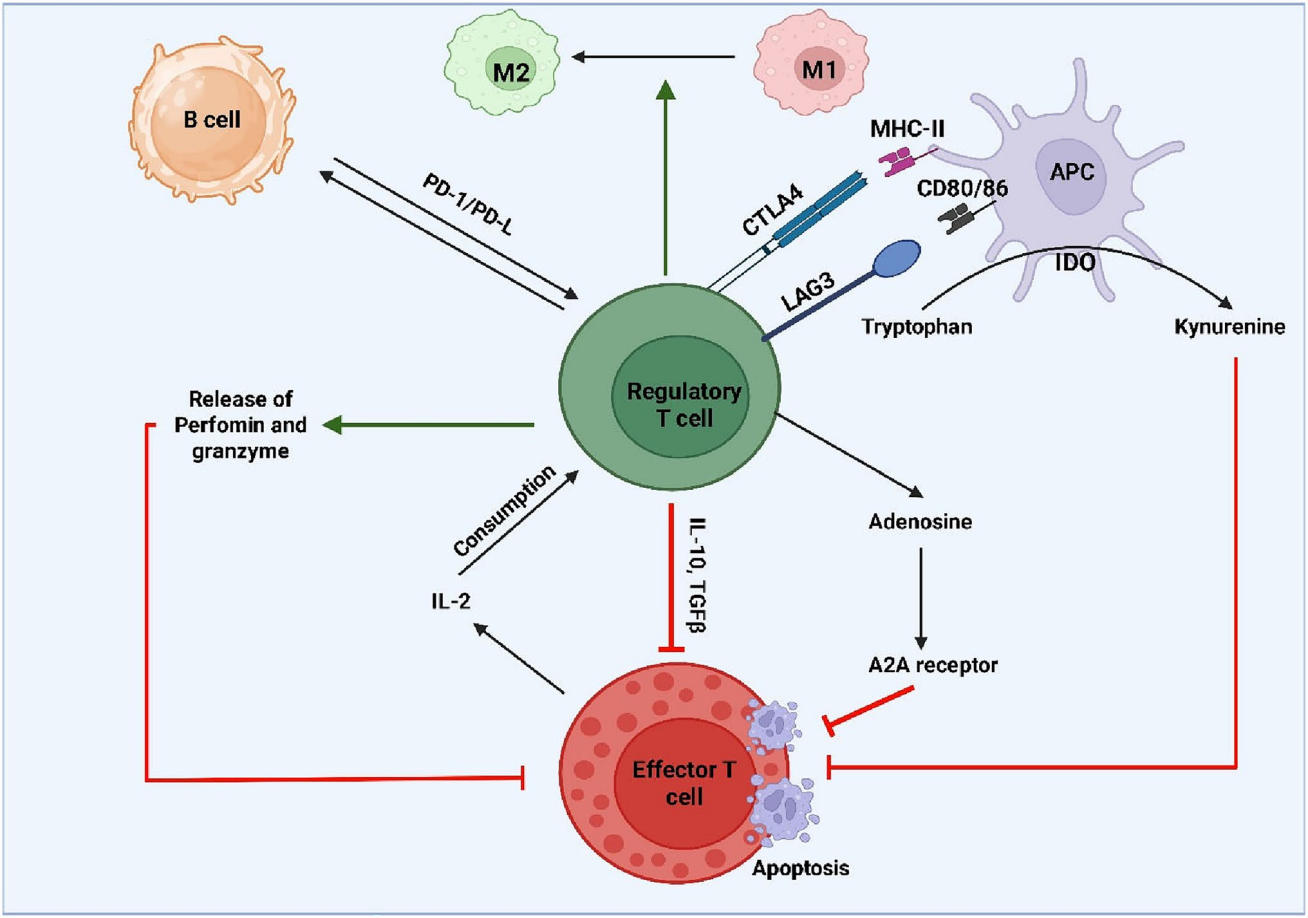
Mechanisms of Treg suppression. This figure illustrates the multifaceted ways Treg cells exert their immunosuppressive effects. Tregs interact with Antigen-Presenting Cells (APCs) via Cytotoxic T-Lymphocyte-Associated Protein 4 (CTLA-4) and Lymphocyte Activation Gene-3 (LAG-3), leading to the downregulation of co-stimulatory molecules (CD80/86) and activation of Indoleamine 2,3-Dioxygenase (IDO), which catabolizes tryptophan into kynurenine, inhibiting Effector T cell proliferation. Tregs also consume Interleukin-2 (IL-2), depriving Effector T cells of a vital growth factor, and release immunosuppressive cytokines like Interleukin-10 (IL-10) and Transforming Growth Factor-beta (TGF-β). Furthermore, Tregs can induce apoptosis in Effector T cells through perforin and granzyme release, and produce adenosine, which binds to A2A receptors on Effector T cells, mediating suppression. Tregs also influence macrophage polarization (M1 to M2) and B cell function via PD-1/PD-L

Tregs can suppress effector T cells by inducing a metabolic state that is not permissive (Munn et al. 2018). Their inherent high expression of CD25 (IL-2Rα), which enables them to metabolize and ingest ambient IL-2, is one of the main mechanisms. Tregs’ use of IL-2 efficiently deprives conventional T cells of growth and effector function because it is an essential growth factor for effector T cell development and survival (Amit et al. 2023). Additionally, Tregs may be ectoenzyme-positive for CD39 and CD73, which hydrolyze ATP and ADP to adenosine (Allard et al. 2020). In turn, adenosine further reduces the immune response by activating intracellular signaling pathways that result in immunosuppression by activating adenosine A2A receptors on effector T cells and APCs. Additionally, Tregs can limit effector T cell growth by passing cAMP to effector T cells in gap junctions (Allard et al. 2020) (Fig. 2).

### Tregs in specific neuroinflammatory and neurodegenerative diseases

Immune homeostasis is based on the delicate balance between effector T cells and Tregs; any disruption, particularly a lack of Treg function or quantity, can cause or worsen a range of neuroinflammatory and NDDs (Zhang et al. 2024). Therefore, improving or restoring Treg activity has become an intriguing immunomodulatory approach to many incapacitating disorders.

Treg dysfunction or deficiency is a known disease pathogenic factor in MS, an inflammatory illness marked by persistent inflammation, demyelination, and neurodegeneration in the CNS. Reduced circulating levels of Tregs or, more commonly, dysregulated suppressive function of Tregs are common in MS patients, particularly those experiencing active relapses (Venken et al. 2010). This disrupts control, enabling pathogenic Th1 and Th17 cells to proliferate unchecked, penetrate the weakened BBB, and initiate an autoimmune attack on CNS myelin structures and possibly neurons (Venken et al. 2010). Treg stability and function may potentially be disrupted by the inflammatory environment in MS lesions, resulting in an inflammatory loop Genetic predisposition (e.g., HLA-DR15), epigenetic changes, and exposure to pro-inflammatory cytokines (e.g., IL-6, IL-1β) that might induce Treg plasticity towards a Th17-like destiny are some of the pathways leading to Treg dysfunction in MS (Hu et al. 2024). Thus, methods for increasing the quantity or suppressive capacity of Tregs are being researched as possible treatments for MS.

Beyond autoimmune diseases, Tregs play a crucial role in protective roles in models of acute CNS injury from stroke and TBI, where uncontrolled inflammation greatly increases the risk of secondary brain damage and poor functional result (Hey et al. 2023). Tregs actively suppress the immediate inflammatory response after migrating into the ischemic brain parenchyma after an ischemic stroke (Hey et al. 2023). They accomplish this by releasing anti-inflammatory cytokines like as TGF-β and IL-10, which inhibit the production of pro-inflammatory mediators, stop neutrophil and pathogenic T cell invasion, and inhibit microglial activation (Harkins et al. 2022). In stroke and TBI models, Tregs have been demonstrated to do more than only reduce inflammation; they also support neurovascular remodeling, angiogenesis, and neurogenesis, ultimately aiding in brain healing and function restoration (Harkins et al. 2022). Although the function of Tregs in neurodegenerative long-term disorders such as PD and AD is more complicated, there is significant therapeutic potential for restoring Treg activity. With some findings of decreased Tregs or a malfunction in their suppressive function within the peripheral and central nervous systems of AD patients, there is mounting evidence of an impaired immune balance in AD. This leads to persistent neuroinflammation and the worsening of tau and Aβ pathology (MacHhi et al. 2020). By releasing neurotrophic factors or anti-inflammatory cytokines, Tregs may reduce neuronal damage, improve the clearance of pathologic protein aggregates, and modify microglial activation states. Tregs and pathogenic T cells (such Th17 and CD8+) are pathologically out of balance in PD (MacHhi et al. 2020). The neuroinflammatory milieu that promotes dopaminergic neuronal death is exacerbated by the relative loss or malfunction of Tregs (MacHhi et al. 2020).

## Therapeutic strategies modulating T-cell responses in CNS disorders

### General immunosuppressive/immunomodulatory agents

The cornerstone of care for life-threatening autoimmune and neuroinflammatory disorders has long been traditional immunosuppressive and immunomodulatory therapies, which are frequently crucial in reducing acute flare-ups or managing the activity of chronic diseases (Durcan and Petri 2016). They usually have broad, non-selective immune-system-affecting mechanisms that impair immune function, generally without targeting particular disease pathways (Durcan and Petri 2016). Because of their strong anti-inflammatory and immunosuppressive qualities, corticosteroids, like methylprednisolone and prednisone, are perhaps the most commonly utilized. Their typical mode of action involves binding to intracellular glucocorticoid receptors, which then go into the nucleus to alter gene expression, most notably by inhibiting transcription factors that promote inflammation (Schijvens et al. 2019). Numerous immune cell activities are profoundly immunosuppressed as a result, including decreased production of pro-inflammatory cytokines (IL-1β, IL-6, TNF-α, and IFN-γ), inhibition of leukocyte migration, and induction of lymphocyte death (Schijvens et al. 2019). By lowering edema and inflammatory infiltrates, corticosteroids are essential for quickly downregulating acute exacerbations of neuroinflammation, such as those that occur during MS relapses or severe neuroinflammatory syndromes after stroke or TBI (Sloka and Stefanelli 2005).

Long-term systemic use has been linked to a wide range of debilitating side effects, such as blunting of the hypothalamic–pituitary–adrenal axis, metabolic disturbances (diabetes, obesity), cardiovascular disease (hypertension, atherosclerosis), bone loss (osteoporosis), increased susceptibility to opportunistic infections, and neuropsychiatric effects (mood changes, cognitive impairment). These strict limitations typically preclude long-term use and necessitate thorough risk–benefit analysis (Tavares et al. 2023).

Cytotoxic drugs like methotrexate, cyclophosphamide, and azathioprine are examples of other broad-spectrum immunosuppressants that were initially used for organ transplants or serious autoimmune diseases (Silvis 2001). These medications prevent the proliferation of rapidly dividing cells, such as lymphocytes, by interfering with DNA synthesis or alkylating DNA (Silvis 2001). Despite their ability to suppress immunity, these agents’ non-specific cytotoxic activity is the root cause of serious systemic toxicities, including alopecia, widespread gastrointestinal distress, bone marrow suppression (leukopenia, anemia, and thrombocytopenia), and an increased risk of opportunistic infections and secondary cancers (Silvis 2001). For example, cyclophosphamide is a highly potent immunosuppressant that is used to treat aggressive forms of autoimmune encephalitis, but it is also associated with high-risk infertility and hemorrhagic cystitis (Osen et al. 2023). Similarly, calcineurin inhibitors, such as tacrolimus and cyclosporine A, work by blocking the activity of calcineurin, a phosphatase that is essential for activation of nuclear factor of activated T cells (Chen et al. 2022). This stops the transcription of the IL-2 gene and, consequently, T-cell activation and proliferation (Chen et al. 2022). Their usage in neuroinflammatory disorders is severely limited due to extremely unpleasant side effects, such as nephrotoxicity, hypertension, neurotoxicity (tremor, seizures), and infection susceptibility, even though they are quite successful at preventing transplant rejection (Chen et al. 2022). The limited treatment window caused by this non-selective approach emphasizes the critical need for new selective immunomodulatory techniques that may precisely control pathogenic T-cell responses without impairing beneficial immunological activities.

### Specific blockade of pathogenic T-cell responses

More so than broad-spectrum immunosuppression, which has serious systemic adverse effects, the development of highly focused immunotherapies marks a significant advancement in the treatment of neuroinflammatory and autoimmune diseases (Yasmeen et al. 2024). To improve efficacy and maybe reduce bystander harm to the immune system as a whole, these techniques seek to specifically block particular pathways or cell types that are considered harmful (Yasmeen et al. 2024).

Monoclonal antibodies (mAbs) against T-cell surface antigens are one of the well-known strategies; they can either deplete subsets of T-cells or inhibit their function (Martin et al. 2013). The highly efficient humanized mAb alemtuzumab (anti-CD52) binds to CD52, a glycoprotein that is abundant on monocytes and lymphocytes (T and B cells). These cells are rapidly, severely, and permanently removed from the circulation as a result of this binding (Jordan et al. 2005). This is followed by a typical immune reconstitution process that takes place over months to years, usually resulting in a less inflammatory and more regulatory immunological profile (Jordan et al. 2005). Careful patient selection and ongoing monitoring are necessary because of the significant risks associated with its powerful effects, which include infusion reactions, the development of secondary autoimmune diseases (such as thyroid disease and immune thrombocytopenia), and an increased vulnerability to potentially fatal infections (Brahmer et al. 2018). Rituximab (anti-CD20), a B-cell-depleting medication, indirectly modulates T-cell activation in MS by reducing B-cell-mediated antigen presentation and cytokine release. This reflects the intricate relationships between immune cell subsets (Kamburova et al. 2013).

Another option under investigation is teplizumab (anti-CD3), an anti-CD3 humanized monoclonal antibody that targets the CD3 complex on T cells (Kokori et al. 2024). Its mechanism involves either the production of regulatory T cells or the creation of partial T-cell depletion and T-cell anergy (Kokori et al. 2024). Although more widespread systemic T-cell depletion and cytokine release syndrome are feared, teplizumab has shown promise in preventing the clinical onset of type 1 diabetes and may be used as an early treatment for other T-cell-mediated autoimmune diseases, such as those affecting the CNS (Kokori et al. 2024).

Although they are mostly used to treat psoriasis and ankylosing spondylitis, anti-IL-17 drugs like eculizumab and afelimomab particularly target IL-17A, a major cytokine generated by Th17 cells that has tissue-ruinous and highly inflammatory effects in a range of autoimmune diseases (Țiburcă et al. 2022). Since Th17 cells play a major role in the pathophysiology of MS, anti-IL-17 therapy offers great preclinical potential for treating neuroinflammatory illnesses by preventing neutrophil infiltration, reducing tissue damage, and maybe repairing the integrity of the BBB. (Shi et al. 2022). However, overcoming BBB penetration and potential systemic adverse effects, such as an elevated risk of mucocutaneous candidiasis, will be necessary for successful therapeutic use in the CNS (Shi et al. 2022).

Finally, therapies that block T-cell trafficking to the CNS have proven to be very successful (Tanasescu et al. 2023). One excellent example is natalizumab (anti-α4-integrin), a humanized monoclonal antibody that specifically targets the α4-subunit of integrins (VLA-4) found on the surface of most leukocytes, such as T cells, B cells, and monocytes (Tanasescu et al. 2023). Natalizumab stops these immune cells from transmigrating across the blood–brain barrier and into the CNS parenchyma by blocking the interaction of α4-molecules with VCAM-1 on endothelial cells (Tanasescu et al. 2023). In very active relapsing–remitting MS, this step has made natalizumab extremely effective in lowering the recurrence rate and postponing the development of disability. However, natalizumab carries a significant risk of progressive multifocal leukoencephalopathy, an uncommon but frequently fatal opportunistic JCV brain infection, due to its impairment of CNS immune surveillance (Tanasescu et al. 2023).

An oral medication called fingolimod, an S1P receptor modulator, works by trapping lymphocytes in the lymph nodes and preventing them from leaving and returning to the afflicted regions, including the CNS (Groves et al. 2013). Although it works very well for MS, there are hazards associated with it, including macular edema, bradycardia, higher infection rates, and the possibility of rebound disease after stopping the medication (Groves et al. 2013). More recent generation S1P modulators, such as siponimod and ozanimod, are more selective and less likely to cause side effects. Despite their many advantages over non-selective immunosuppression, such focused methods still have a balance between efficacy and certain safety issues that need to be properly addressed in clinical settings (Yang et al. 2024).

### Antigen-specific immunotherapy

A paradigm shift in the management of autoimmune illnesses, antigen-specific immunotherapies seek to create a particular immunological tolerance against autoantigens without causing the general immunosuppression linked to traditional treatments (Afshari et al. 2025).

One common multirelapse MS medication is glatiramer acetate (Copaxone), which is a four-amino acid random copolymer of myelin basic protein (Simpson et al. 2003). It is thought to function as a “decoy” antigen, triggering a Th2-biased or regulatory T-cell response (such as the production of IL-10 and TGF-β), which then cross-reacts with myelin antigens to suppress the pathogenic Th1/Th17 responses; however, the precise mechanism is complicated (Simpson et al. 2003). Another antigen-specific immunotherapy concept is T-cell vaccination, which involves administering inactivated or attenuated clones of the disease-causing T cells to elicit an anti-idiotypic immune response (Liu et al. 2022).

Inducing CTLs or Treg that precisely target and downregulate the original disease-causing T-cell clones is the aim. This approach has been tried in early-stage clinical trials for rheumatoid arthritis and MS, but it is difficult to define which T-cell clones are truly pathogenic, their heterogeneity, and the difficulties in producing and delivering such customized cellular therapies (Mueller et al. 2021). To distribute antigens in a tolerogenic setting, encapsulated autoantigens in liposomes or nanoparticles are being produced (Benne et al. 2024). In preclinical models of EAE, Type 1 Diabetes, and other autoimmune illnesses, this “tolerogenic vaccine” strategy showed great promise and successfully stopped or reversed the progression of the disease (Arve-Butler and Moorman 2024).

### Interferon-beta (IFN-β) inhibitors

Interferon-beta (IFN-β) is a type I, or class of cytokines, in the interferon family, which are produced within human bodies and possess strong antiviral and immunomodulatory properties (Farhangian et al. 2024). For its place as a treatment for the condition of MS, its mechanisms of action is complicated and multifaceted, yet it aims to bring the immune system back into balance against the hyperactive response that ultimately drives the disease (Farhangian et al. 2024). One important mechanism of activity of IFN-β is its immunosuppressive ability to inhibit T cell proliferation and activation. T cells are one of the Suffered immune players that are ultimately responsible for the pathogenesis of MS (Kasper and Reder 2014). IFN-β changes co-stimulatory molecule expression on APCs and inhibits the APCs' ability to present antigen to T lymphocytes, which is a major control mechanism of T cell activation (Kasper and Reder 2014). Additionally, it can affect the cytokine environment by shifting away from Th1 and Th17, which leads to inflammatory demyelination and neuronal damage to a Th2 type state, which limits inflammation. This shift assists with restoring the immunosuppressive function of Tregs, which are often described as being depleted or ineffective in MS patients (Kasper and Reder 2014). IFN-β downregulates adhesion molecules, like the VLA-4 found on T cells, and it can affect the integrity of the BBB in a way that physically limits the entry of such pathogenic lymphocytes (Balasa et al. 2021). In a clinical context, this translates to a decrease in the number of relapses and a slower rate of progression of disease, as illustrated by pivotal trials that demonstrated a significant reduction in the amount of both clinical relapses and new brain lesions on MRIs (Gärtner et al. 2018). The use of IFN-β is associated with common side effects, including flu-like symptoms and injection site reactions, and these side effects can affect patient adherence. In a more significant limitation, a proportion of patients develop neutralizing antibodies, which can decrease the effectiveness of therapy over time (Filipi and Jack 2019). Although there are more recent and powerful disease-modifying therapies available, IFN-β is a well-known and important first-line treatment with long-term safety and efficacy for relapsing forms of MS (Filipi and Jack 2019).

### Janus Kinase (JAK) inhibitors

The category of small molecules called Janus Kinase (JAK) inhibitors influences immune system function through inhibition of a JAK-signal transducer and activator of transcription (STAT) signaling pathway that initiates important intracellular communication. JAK-STAT signaling is initiated by multiple cytokines and growth factors that are typically overproduced in a state of autoimmunity and/or chronic inflammatory diseases (Lv et al. 2024). This signaling occurs when a cytokine docks to its corresponding receptor at the cell surface, activating the associated JAK enzymes (JAK1, JAK2, JAK3, and TYK2), which subsequently phosphorylate the associated cytokine receptor, which then leads to the phosphorylation of molecules known as STATs. Phosphorylated STAT proteins form dimers, translocate from the cytoplasm to the nucleus, and then act as transcription factors that govern the expression of genes associated with inflammation, immunity, and cell proliferation (Lv et al. 2024). By employing JAK inhibitors to inhibit one or more of the JAK enzymes, JAK inhibition has the capacity to block the cascade of events leading to signaling and the activation of pro-inflammatory cytokines. Ultimately, using JAK inhibitors has the potential to limit the aberrant action of the immune system that is associated with pathogenesis that activates this signaling pathway (Lv et al. 2024).

Research into JAK inhibitors is their growing therapeutic use for chronic inflammatory and autoimmune diseases (Shawky et al. 2022). Currently, JAK inhibitors are approved for the treatment of rheumatoid arthritis, psoriatic arthritis, ulcerative colitis, and atopic dermatitis (eczema) (Shawky et al. 2022). They are also being used on a promising basis to treat alopecia areata by preventing the immune attack of hair follicles and vitiligo, which decreases triphasic repigmentation (Zhou et al. 2024). While some traditional biologics are designed to specifically target one cytokine, JAK inhibitors can inhibit the signaling of multiple cytokines that share the same JAK enzyme and therefore have the potential to provide a broader anti-inflammatory effect (Chikhoune et al. 2025). Although JAK inhibitors are effective, there are significant safety considerations. Because of their immunomodulatory effects, there is an increased risk for serious infections, including serious respiratory tract infections, and the reactivation of latent viruses like herpes zoster (shingles) and tuberculosis (Konzett et al. 2025). There are also concerns regarding the increased risk of Major Adverse Cardiovascular Events, malignancies (especially non-melanoma skin cancer and lymphomas), and blood clots, including pulmonary embolism and deep vein thrombosis. High-risk populations, especially over the age of 65, have a higher risk for all of these side effects, especially if the patient has any past cardiovascular disease or is a current or past long-term smoker (Konzett et al. 2025). Thus, the use of JAK inhibitors must include appropriate patient selection and patient monitoring to ensure the balance between therapeutic benefit and risk.

### Anti-TNF-alpha

Tumor Necrosis Factor-alpha (TNF-α) is a strong pleiotropic cytokine that is a key mediator of the complex inflammatory response. In the context of CNS inflammatory diseases, TNF-α has a complex and often detrimental role in the chronic inflammatory process and in the breakdown of the important BBB (Gonzalez Caldito 2023). TNF-α is produced by a variety of cells, including activated T cells, microglia, and astrocytes. TNF-α can signal through two distinct receptors, TNFR1 and TNFR2. In the case of neuroinflammation, the TNFR1 pathway is primarily viewed as pro-inflammatory and pro-apoptotic, resulting in neuronal injury and exacerbating the disease. TNF-α also directly mediates disruptions in the BBB because it can cause a reorganization of tight junction proteins in brain endothelial cells, leading to increased vascular permeability that allows more infiltrating inflammatory cells, including T cells and other leukocytes, into the CNS. This results in a self-perpetuating cycle; a leaky BBB allows additional immune cell infiltration into the CNS, the immune cells produce more TNF-α, and the leaky BBB becomes worse. As mentioned previously, the cytokine can also directly activate microglia, pushing them into a pro-inflammatory phenotype (Gonzalez Caldito 2023; Shokr et al. 2025; Alshahrani et al. 2025). The activation of microglia can lead to the release of other pro-inflammatory mediators, including IL-1β and IL-6, which further intensify only the neuroinflammatory cascade (Khowdiary et al. 2025; Kamila et al. 2025).

Therapies focused on TNF-α, such as infliximab and adalimumab, neutralize this cytokine and disrupt this inflammatory feedback loop. By binding both the soluble and transmembrane forms of TNF-α, the goal is to suppress the microglial activation leading to protective effects at the BBB and limiting T-cell migration into the CNS (Gonzalez Caldito 2023). While these therapies have shown remarkable success in other autoimmune diseases such as rheumatoid arthritis and inflammatory bowel disease, they have posed a complex and at times paradoxical challenge clinically in the setting of CNS demyelinating diseases such as MS, with some studies suggesting these therapies may even worsen MS (Lopetuso et al. 2023). One potential explanation for this outcome is the duality of TNF-α signaling, in which the blockade of TNFR2, which is involved in myelination and tissue repair, could outweigh the blockade of the pro-inflammatory nature of TNFR1 (Faustman et al. 2025). Thus, ongoing research into developing more specific anti-TNF-α therapies that exclusively target the TNFR1 pathway is an important area of study in CNS inflammatory diseases.

### Sphingosine-1-phosphate (S1P) receptor modulators

Sphingosine-1-phosphate (S1P) receptor modulators are a class of immunomodulatory medication notably symbolized by fingolimod, to treat neuroinflammatory conditions including MS (Coyle et al. 2024). These medications have a unique mechanism of action that is focused on disrupting the normal trafficking of T lymphocytes that amplify autoimmune inflammation in the CNS (Coyle et al. 2024). The mechanism begins with the fact that T cells must escape the lymphoid organs (like lymph nodes) and enter the systemic circulation to surveil the body. The process of egress is tightly controlled by a naturally occurring lipid, S1P, which is present at high concentration in the plasma circulation but at extremely low concentration in the lymph node parenchyma. T cells in the lymph node express S1PR1, a surface receptor that can register the S1P extracellular gradient (Maeda et al. 2010). The S1P receptor modulators are small molecules which will activate the S1PR1 receptor but then bind to it. The binding not only inhibits functional activity but also induces internalization and degradation of S1PR1. When T cells do not express S1PR1 on their surface receptor, they lose the ability to sense and stay oriented with the S1P gradient and effectively get trapped in the lymph node (Maeda et al. 2010). This sequestration dramatically decreases the number of pathogenic T cells circulating through the blood and, just as importantly, it stops those T cells from crossing the blood–brain barrier to penetrate the CNS (Maeda et al. 2010). By inhibiting this penetration of the CNS, these drugs can reduce the inflammatory attack on myelin sheath and neurons, which will decrease the number of relapses while slowing the progression of disability in MS patients (Cohan et al. 2020). The target-specificity of this therapy, based on lymphocyte trafficking rather than broad immunosuppressive effects, represents a fantastic option for controlling these chronic diseases (Cohan et al. 2020).

### New directions

Growing knowledge of immune cell biology, CNS-immune interactions, and sophisticated biotechnological tools is driving a rapid evolution in the therapy landscape for neurodegenerative and neuroinflammatory illnesses. Novel biological mechanisms and precision engineering are being used in emerging areas to provide highly focused and effective immunomodulation in order to restore CNS homeostasis without causing widespread immunosuppression (Toader et al. 2024).

The influence of microbiome modification on T-cell responses is one such exciting avenue. The gut-brain axis explains how the gut microbiota significantly affects systemic and CNS immunity (Ashique et al. 2024). Short-chain fatty acids (SCFAs) like butyrate, propionate, and acetate are among the many metabolites produced by gut microorganisms that may have a direct impact on immune cell development and function. Butyrate, for example, can enhance pro-inflammatory Th1 and Th17 responses that contribute to neuroinflammation by promoting the differentiation and suppressive function of Tregs both in the gut and throughout the body (Qu et al. 2023). On the other hand, dysbiosis, or an imbalance in the gut microbiota, has been linked to the aggravation of neuroinflammatory diseases such as AD, PD, and MS by, at least in part, permitting the invasion of the CNS by pathogenic T cells (Ashique et al. 2024). To reconstitute the gut microbiota and guide T-cell responses towards a more regulatory and neuroprotective phenotype, treatment options include fecal microbiota transplantation, targeted probiotic or prebiotic treatment using well-characterized bacterial strains or consortia, or direct SCFA administration (Sahle et al. 2024). Stable engraftment of beneficial microorganisms, determining the exact molecular linkages, and the dynamic and heterogeneous character of the human microbiome present challenges. T cell metabolic reprogramming also presents a potential window of opportunity (Sahle et al. 2024).

Their metabolism is tightly linked to T-cell activation, effector function, and differentiation. For example, Tregs often prefer oxidative phosphorylation to ensure suppressive activity, whereas pro-inflammatory and rapidly multiplying effector T cells (Th1, Th17) would typically use aerobic glycolysis (Fiorucci et al. 2021). This differential metabolic reliance creates therapeutic opportunities: treatments could specifically block pathologic T-cell proliferation and effector function or improve Treg differentiation and suppressive capabilities by targeting specific metabolisms (Kim et al. 2025). According to preclinical research, T-cell metabolism modification may be useful in the CNS and has a major effect on the onset of autoimmune diseases (Ma et al. 2024). It can be difficult to guarantee specificity and prevent unintended metabolic changes to other cells. The rapidly developing science of non-coding RNAs (ncRNAs), including long non-coding RNAs (lncRNAs) and microRNAs (miRNAs), offers new opportunities for immunomodulation (Zong et al. 2023). These ncRNAs have a significant effect on T-cell differentiation, activation, and function, but they do not code for proteins; instead, they control gene expression post-transcriptionally (Taheri et al. 2021).

## Limitations and future perspectives

Notwithstanding the remarkable progress made in elucidating the dynamic roles of T-cell subsets in NDDs, a number of formidable obstacles and constraints still need to be addressed, which will determine the vital future lines of inquiry. The intrinsic complexity and plasticity of T-cell subsets themselves present one of the main obstacles; their phenotypes and roles are extremely context-dependent, disease-dynamic as it progresses, and controlled by the complex CNS microenvironment. It is still difficult to distinguish between truly pathogenic and protective or bystander T cells in vivo, especially in humans with restricted access to the CNS. The majority of therapeutic compounds, such as cell treatments or big mAbs, cannot be delivered via the CSF barrier, which hinders the creation of innovative delivery methods. Furthermore, the discovery of genuinely antigen-specific immunotherapies is delayed by the imprecise designation of the specific autoantigens that trigger the T-cell response in the majority of NDDs. A one-size-fits-all immunomodulatory strategy is implausible due to the variety of NDDs themselves, which have multiple etiologies and pathologies. This emphasizes the necessity of personalized medical approaches. Direct preclinical translatability is limited by the tendency of current animal models to only partially replicate the chronicity and complexity of human NDDs. Furthermore, it is difficult to stratify patients, evaluate therapy response, and detect diseases early due to the absence of reliable, readily accessible biomarkers for tracking T-cell function and inflammatory states of the human CNS.

Given how quickly technology is developing, the future seems really bright here. In order to define new pathogenic or regulatory populations and to unravel the distinct molecular and functional status of T-cell subsets in the CNS at hitherto unheard-of resolution, single-cell multi-omics platforms, such as single-cell RNA sequencing, TCR sequencing, and epigenomics, will be crucial. This will make it possible to find new, extremely targeted treatment targets. Advanced neuroimaging techniques will enable longitudinal, non-invasive monitoring of T-cell activity and neuroinflammation in vivo. Since altering these connections has significant therapeutic potential, research will increasingly examine the two-way communication between T cells and glial cells that reside in the CNS, such as microglia and astrocytes. To improve CNS penetration in biologics and cell treatments, new delivery mechanisms (such as targeted ultrasound, tailored nanoparticles, and viral vectors) must be developed. Additionally, combinatorial strategies that combine T-cell regulation with therapies aimed at other immune cells, metabolic pathways, or even proteinopathy-directed medicines are probably going to be a part of next-generation therapeutics. The combination of artificial intelligence and machine learning will help decipher intricate immunological data to forecast disease progression and improve treatment plans. This will pave the way for highly customized immunotherapies that accurately restore CNS immunity, maximize neuroprotection, and eventually stop or reverse neurodegeneration.

## Conclusion

With their incredibly diverse roles, T lymphocytes are becoming more and more acknowledged as important participants in the intricate relationship between immunomodulation and neuroinflammation in the CNS. The onset, progression, and recovery from neuroinflammatory and neurodegenerative diseases are greatly influenced by adaptive immune cells, ranging from the pathogenic Th1, Th17, and CD8+ T cells’ destructive actions that promote neuronal damage and demyelination to the crucial neuroprotective and immunosuppressive functions of regulatory T cells. It is crucial to clarify the exact processes by which T-cells enter the CNS, how they interact with resident glial cells, and the distinct molecular pathways that govern their activation and differentiation. Unprecedented difficulties arise with the transition from broad immunosuppression to highly particular immunomodulatory techniques such as cytokine neutralization, monoclonal antibodies, and the newer areas of cellular engineering and antigen-specific immunotherapies. Future treatments hold great potential to stop or even reverse the debilitating progression of these crippling neurological conditions by precisely regulating T-cell responses and boosting defensive immunological functions.

## Data Availability

All data used for the review article have been cited in the text.

## Abbreviations

AD: Alzheimer’s disease
APC: Antigen-presenting cell
Aβ: Amyloid-beta
BBB: Blood–brain barrier
BCSFB: Blood-cerebrospinal fluid barrier
CD4+: Cluster of differentiation 4
CD8+: Cluster of differentiation 8
CNS: Central nervous system
CSF: Cerebrospinal fluid
CTLA-4: Cytotoxic T-lymphocyte-associated protein 4
DC: Dendritic cell
EAE: Experimental autoimmune encephalomyelitis
FasL: Fas ligand
FoxP3: Forkhead box P3
IFN-γ: Interferon-gamma
IL-1β: Interleukin-1 beta
IL-10: Interleukin-10
IL-17: Interleukin-17
MHC: Major histocompatibility complex
MMP: Matrix metalloproteinase
MS: Multiple sclerosis
NDD: Neurodegenerative disease
PD: Parkinson’s disease
PD-1: Programmed death-1
SCFA: Short-chain fatty acid
TCR: T-cell receptor
TGF-β: Transforming growth factor-beta
Th1: T helper 1 cell
Th17: T helper 17 cell
TJ: Tight junction
TNF-α: Tumor necrosis factor-alpha
Treg: Regulatory T cell
VCAM-1: Vascular cell adhesion molecule-1
VLA-4: Very late antigen-4
